# Obstructive jaundice with duodenal biliary fistula: diagnostic and therapeutic challenges, case report, and literature review

**DOI:** 10.3389/fmed.2025.1644045

**Published:** 2025-09-26

**Authors:** Rongchun Xing, Yiwei Hou, Li Yi, Qingjin Wan, Guoxin Chen, Yashan Wang, Mingzheng Hu, Yu Yang

**Affiliations:** ^1^The First College of Clinical Medical Science, China Three Gorges University, Yichang, Hubei, China; ^2^Department of Hepatobiliary Surgery, Yichang Central People’s Hospital, Yichang, Hubei, China; ^3^Department of Endocrinology, Yichang Central People’s Hospital, Yichang, Hubei, China; ^4^Medical Technology College of Qiqihar Medical College, Qiqihar, Heilongjiang, China

**Keywords:** case report, obstructive jaundice, bile duct stones, biliary-enteric fistula, endoscopic retrograde cholangiopancreatography, laparoscopic surgery

## Abstract

**Background:**

This case presents a 56-year-old male with obstructive jaundice, complicated by bile duct stones, bile duct dilation, and a possible biliary-enteric fistula. This case contributes to the literature by highlighting the complexities of managing obstructive jaundice in patients with complex surgical histories.

**Case summary:**

The patient, with a history of laparoscopic cholecystectomy (2022), presented with upper abdominal pain, jaundice, and fever. Imaging (ultrasound, CT, MRCP) revealed intrahepatic bile duct dilation, pneumobilia, stones, and a biliary-enteric fistula. Initial ERCP attempted fistula dilation but was unsuccessful due to inflammatory fibrosis. Subsequent laparoscopic partial gastrectomy with Roux-en-Y gastrojejunostomy resolved the obstruction. Postoperative recovery was uneventful, with resolution of jaundice and scheduled six-month follow-up.

**Conclusion:**

This case highlights the critical role of multimodal imaging (MRCP/CT) in diagnosing rare biliary-enteric fistulas, particularly in patients with surgical histories. A multidisciplinary approach—combining endoscopic intervention and tailored laparoscopic surgery—proved effective for managing complex obstructive jaundice. Vigilant postoperative surveillance is essential to mitigate recurrence risks. Future studies should explore minimally invasive techniques for similar cases.

## 1 Introduction

This case report presents a 56-year-old male patient diagnosed with obstructive jaundice, complicated by bile duct stones, bile duct dilation, and a possible biliary-enteric fistula. The patient’s medical history included previous abdominal surgeries, such as laparoscopic cholecystectomy and intestinal adhesion release, which likely predisposed him to these complications. Despite initial supportive treatment, his condition progressed, requiring invasive interventions like endoscopic retrograde cholangiopancreatography (ERCP) and laparoscopic surgery to address the biliary obstruction (1).

This case is noteworthy due to the complex interplay of factors, including the rare complication—a biliary-enteric fistula—likely resulting from prior surgery (2). It highlights the challenges in diagnosing and treating obstructive jaundice in patients with a complex surgical history and underscores the importance of a multidisciplinary approach.

Our literature review examined diagnostic approaches and treatments for obstructive jaundice complicated by surgical history, using key terms including “biliary-enteric fistula” and “post-surgical biliary complications.” While biliary-enteric fistulas are rare (3), they occur in patients with prior biliary surgeries (Table 1). This case contributes to understanding biliary disease management in complex clinical histories, emphasizing early detection, monitoring, and tailored intervention.

**Table 1:** Post-cholecystectomy biliary-enteric fistula with stone-related obstructive jaundice: chronological case reports.

Title	First author and year	Presentation	Management	Outcomes/lessons	Critical insights and synthesis
Management dilemma of cholecysto-colonic fistula: Case report	Gibreel W (2018) (7)	77-year-old male with progressive abdominal distension, diarrhea, biliary obstruction, and septic shock	Urgent open partial cholecystectomy, segmental colonic resection (failed ERC biliary decompression)	Resolution of sepsis; highlights urgency in managing biliary obstruction with fistulae	Cholecystocolonic fistula post-cholecystectomy requires prompt intervention if biliary decompression fails to avert biliary sepsis. Conservative management is insufficient if obstruction persists
Gallstone ileus: Unfamiliar cause of bowel obstruction. Case report and literature review	Hussain J (2018) (18)	88-year-old female with small bowel obstruction; history of cholecystectomy. CT showed bilioenteric fistula and jejunal gallstone	Laparotomy, enterolithotomy (without fistula manipulation due to severe adhesions)	Uneventful recovery; symptom-free at 1-year follow-up	Enterolithotomy alone suffices in high-risk patients with post-cholecystectomy gallstone ileus; fistula repair is not mandatory acutely. Diagnostic delay increases morbidity
Spontaneous choledochoduodenal fistula in a patient with a bile duct injury following laparoscopic cholecystectomy	Gallagher SP (2019) (19)	67-year-old female with CBD/right hepatic artery injury post-LC; external biliary catheter-induced fistula	Fistula diagnosed during follow-up; managed conservatively? (Details incomplete)	Illustrates catheter erosion as a rare fistula etiology post-BDI	Iatrogenic bile duct injury complications (e.g., external drains) can lead to secondary fistulae. Long-term monitoring is essential
Robotic-assisted completion cholecystectomy with repair of cholecystoduodenal fistula	Hurwitz JC (2023) (20)	Post-cholecystectomy syndrome due to retained gallbladder remnant; cholecystoduodenal fistula	Robotic completion cholecystectomy, fistula repair	Successful resolution; emphasizes robotic utility in reoperative complex anatomy	An incomplete cholecystectomy risks fistula formation. Robotic approach feasible for fistula closure in reoperations, offering precision in hostile fields
Incidentally Found Cholecystoduodenal Fistula and an Unusual Case of Gallstone Ileus After Laparoscopic Cholecystectomy	Alsairy S (2023) (21)	44-year-old female with post-LC biliary complications; an undiagnosed fistula led to gallstone ileus	Subtotal cholecystectomy, fistula repair (1st surgery); subsequent enterolithotomy for ileus (2nd surgery)	Patient died post-op from multi-organ failure	Key lesson: Intraoperative fistula detection during cholecystectomy is critical. Undiagnosed fistulae cause severe complications (e.g., ileus), increasing mortality
Gallstone ileus: A case treated with minilaparotomy and a review of the literature	Zappia F (2017) (22)	81-year-old female with SBO 25 years post-LC; CT showed pneumobilia, ectopic gallstone	Minilaparotomy, enterolithotomy	Stone extraction successful; fistula not addressed acutely	Gallstone ileus can occur decades post-cholecystectomy. CT is diagnostic (pneumobilia + SBO). Enterolithotomy alone is preferred in elderly/high-risk patients
Interval robotic cholecystoduodenal fistula repair and cholecystectomy for gallstone ileus: A case report	Agathis AZ (2024) (8)	86-year-old female with gallstone ileus post-LC; managed initially with enterolithotomy	Interval robotic cholecystectomy + fistula repair after ileus resolution	Duodenal repair confirmed intact; patient returned to baseline function	Staged management (ileus first, fistula later) is viable. Robotic repair is safe in elective settings for biliary-enteric fistulae post-cholecystectomy

This case exemplifies a post-cholecystectomy biliary-enteric fistula (BEF), aligning with the table’s focus on postoperative fistulae complications (e.g., Gibreel W 2018, Agathis AZ 2024). It highlights shared challenges: ERCP failure necessitating surgery, complex adhesion management, and the critical need for fistula detection to avert severe outcomes like obstruction or sepsis.

## 2 Case presentation

### 2.1 Patient information

The patient is a 56-year-old male, presenting with a complaint of upper abdominal bloating and pain that began a week ago. His medical history includes a previous laparoscopic cholecystectomy and intestinal adhesion release in 2022. The patient has a smoking history of 50 cigarettes per day for 20 years but quit 12 years ago, and he drinks alcohol occasionally. His family history is non-contributory, with no reported hereditary diseases.

### 2.2 Chief complaints and history of present illness

One week before admission, the patient developed persistent dull upper abdominal pain with bloating, nausea, and fever. He was initially treated with gastric protection, spasm relief, and antibiotics at a local hospital, which temporarily alleviated symptoms, but his condition worsened, and one day before presentation, he developed jaundice and dark urine.

### 2.3 Past medical history

The patient denied any history of chronic diseases such as hypertension, diabetes, or coronary heart disease. There was no history of infectious diseases like hepatitis B or tuberculosis. He has a history of urinary tract stones and bile duct dilation. His past surgical history includes a laparoscopic cholecystectomy and intestinal adhesion release performed in November 2022.

### 2.4 Physical examination

Upon admission, the patient’s vital signs were stable: body temperature 36.7 °C, pulse 80 beats per minute, respiratory rate 19 breaths per minute, and blood pressure 143/83 mmHg. The patient was alert and cooperative during the examination. Physical examination revealed jaundice of the skin and sclera. The abdomen was soft and non-tender, with no rebound tenderness or palpable masses. There was no visible abdominal wall varicosity, and the Murphy’s sign was negative. Hepatomegaly or splenomegaly was not detected.

### 2.5 Diagnostic workup

**Figure 1:**
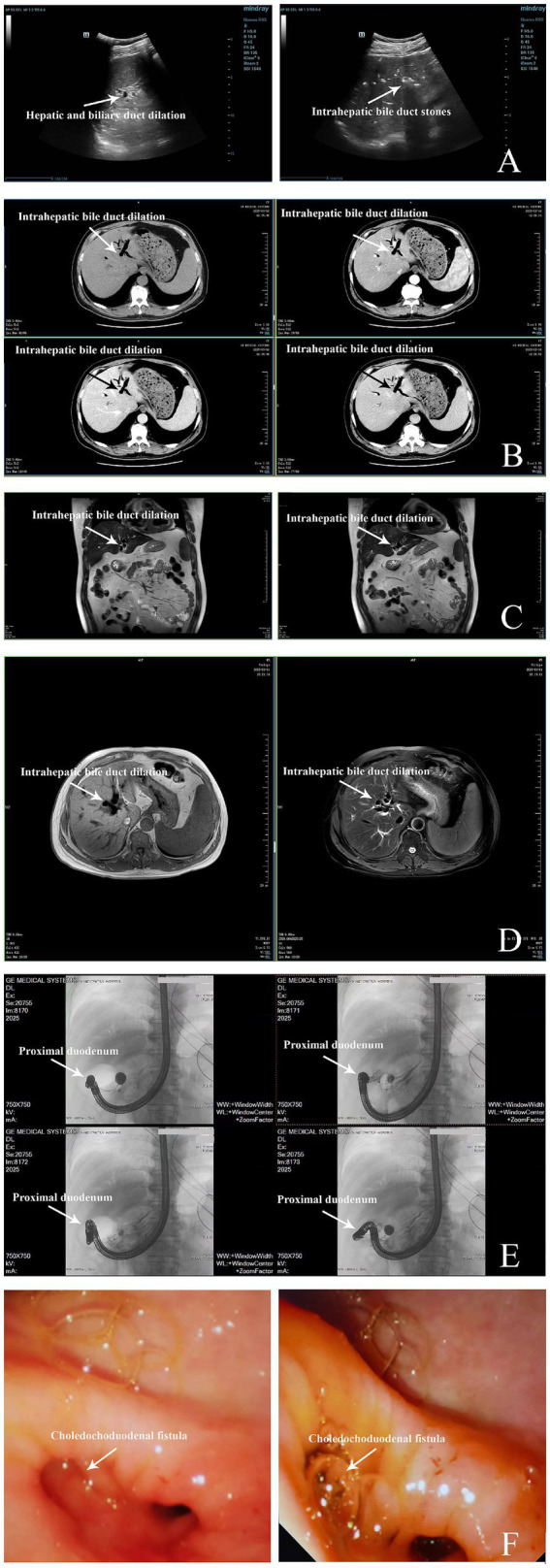
Imaging findings of gallbladder and related conditions. **(A)** Color Doppler ultrasound of hepatobiliary system (7 days before diagnosis): Ultrasound demonstrates heterogeneous hepatic echogenicity with anterior hyperechoic density and posterior attenuation, consistent with mild steatosis. Multiple beaded hyperechoic foci (largest 1.5 × 0.5 cm) with posterior shadowing suggest intrahepatic biliary calculi or pneumobilia. Post-cholecystectomy anatomy with a non-dilated proximal common bile duct (0.5 cm) and splenomegaly (length 12.1 cm, thickness 4.3 cm). Color Doppler flow imaging (CDFI) reveals preserved hepatic and splenic vascular flow, consistent with non-obstructive pathology. **(B)** Contrast-enhanced CT of Hepatobiliary, Pancreatic, and Splenic Systems (7 days before diagnosis): Axial CT demonstrates intra- and extrahepatic biliary pneumobilia and dilation, a non-visualized gallbladder (post-cholecystectomy), hepatic hyperdense nodules, and mild splenomegaly. The pancreas appears unremarkable. Measurements: hepatic nodule size up to 0.5 cm; splenic length ~12 cm. **(C)** Non-contrast MRCP (6 days before diagnosis): Magnetic resonance cholangiopancreatography reveals mild intrahepatic bile duct dilatation with pneumobilia, absent gallbladder (post-cholecystectomy), and splenomegaly. Pancreatic ductal anatomy preserved, no choledochal or pancreatic duct obstruction. **(D)** MRCP (5 days before diagnosis): MRCP confirms intrahepatic bile duct dilation (IHBRD) with pneumobilia, absent gallbladder, non-dilated choledochus and pancreatic duct, and no intraductal filling defects. **(E)** ERCP (on diagnosis date, March 17, 2025): Endoscopic retrograde cholangiopancreatography demonstrates a choledochoduodenal fistula with two inflammatory strictures at the distal duodenum and fistula orifice. Balloon dilation unsuccessful, bile drainage limited. **(F)** Intraoperative Endoscopy (3 days after diagnosis, March 20, 2025): Endoscopic view during laparoscopic surgery reveals choledochoduodenal fistula between the common bile duct and duodenal bulb with an inflammatory stricture and active bile leakage at the fistula site.

Imaging studies, including ultrasound and CT scans, were performed. Abdominal ultrasound revealed irregular distribution of echogenic spots within the liver, suggestive of liver disease with possible bile duct stones or gas accumulation (Figure 1A). The gallbladder was absent due to prior cholecystectomy, and the bile ducts were dilated. A contrast-enhanced CT scan showed similar findings, with the liver demonstrating high-density nodules and bile duct dilation, while the spleen was mildly enlarged (Figure 1B).

The Magnetic Resonance Cholangiopancreatography (MRCP) confirmed intrahepatic bile duct dilation and gas accumulation, consistent with the diagnosis of cholangitis (Figures 1C,D). Imaging findings from ultrasound, CT, and MRCP collectively indicated the presence of bile duct dilation, pneumobilia, and a biliary-enteric fistula. Further diagnostic workup supported the possibility of a fistula secondary to previous surgery or infection.

### 2.6 Diagnosis

The primary diagnosis was obstructive jaundice, likely caused by a combination of bile duct stones and bile duct dilation, with possible biliary-enteric fistula. Other differential diagnoses included hepatobiliary infection and intrahepatic cholestasis.

### 2.7 Prognosis

Based on the imaging findings and the patient’s clinical presentation, the prognosis was cautiously optimistic, but the patient’s condition required close monitoring and further therapeutic intervention.

### 2.8 Therapeutic intervention

Given the severity of the symptoms and the potential complications associated with obstructive jaundice, a multidisciplinary team decided to proceed with endoscopic intervention. The first procedure, performed on March 17, 2025, involved ERCP to attempt dilation and drainage of the bile ducts. The patient was placed under general anesthesia, and an endoscope was inserted to explore the duodenal and biliary structures. During the procedure, a bile duct-duodenal fistula was identified, and balloon dilation was performed (Figure 1E). However, the procedure was complicated by inflammation at the site of the fistula, making it difficult to fully access the bile ducts.

Due to the challenges encountered during ERCP, a second procedure was scheduled for March 20, 2025, involving laparoscopic surgery, which included a partial gastrectomy with Roux-en-Y gastrojejunostomy. The patient was placed under general anesthesia, and the procedure began with the establishment of pneumoperitoneum and insertion of laparoscopic working ports. Dense adhesions between the duodenum, stomach, and liver were identified and carefully dissected to improve access (Figure 1F). After partially resecting the stomach, the jejunum was anastomosed to the remaining stomach and further connected with a Roux-en-Y procedure to bypass the biliary obstruction. The surgery also involved adhesiolysis and the placement of drains at the surgical site. The procedure concluded successfully without immediate complications, and the patient was transferred to the post-operative care unit for recovery (see Table 2 for a chronological overview of all diagnostic and therapeutic steps).

**Table 2:** Chronological summary of diagnostic and therapeutic interventions in a patient with obstructive jaundice and biliary-enteric fistula.

Date	Event	Details
2022/11/19	Prior surgical history	Laparoscopic cholecystectomy + intestinal adhesiolysis performed
2025/3/10	Symptom onset	Developed upper abdominal distension and pain (persistent dull ache), nausea, and fever. Presented to local ER; treated with gastric protection, antispasmodics, and antibiotics. Pain temporarily resolved
2025/3/16	Symptom progression	Worsening abdominal pain, jaundice (scleral icterus), and dark urine. Admitted to the current hospital with suspected obstructive jaundice
2025/3/17	Preoperative imaging	Ultrasound/CT/MRCP Findings: Mild fatty liver, intrahepatic bile duct stones/pneumobilia, biliary dilation, splenomegaly, gallbladder absent (post-cholecystectomy). Suspected biliary-enteric fistula
2025/3/17	ERCP	Attempted endoscopic duodenal/biliary dilation + nasobiliary drainage. Identified duodenal-biliary fistula with inflammatory strictures. Balloon dilation was unsuccessful due to fibrosis
2025/3/20	Laparoscopic surgery	Procedures: Partial gastrectomy + Roux-en-Y gastrojejunostomy + adhesiolysis. Intraoperative findings: Dense adhesions at the hepatoduodenal region, duodenal inflammation, and confirmed biliary-enteric fistula. Post-op drains were placed
2025/3/20	Pathology report	Resected gastric specimen: Chronic mucosal inflammation, no malignancy
2025/3/21	Postoperative imaging (CT)	Mild anastomotic edema, intra-abdominal effusion, persistent biliary dilation/pneumobilia, splenomegaly. No immediate surgical complications
Postoperative	Follow-up	Stable recovery; jaundice resolved. Discharged with plans for diabetes/fatty liver management and long-term biliary monitoring

Timeline outlining key clinical events, imaging findings, surgical interventions, and outcomes (March 10–21, 2025), contextualizing diagnostic challenges and therapeutic progression from symptom onset to postoperative resolution of jaundice.

### 2.9 Follow-up and outcomes

Postoperatively, the patient recovered well and was monitored for any signs of infection or complications. Imaging performed after surgery revealed mild swelling at the surgical sites and the presence of some fluid collections in the abdominal cavity, consistent with normal post-surgical changes. The biliary system showed continued mild dilation, but no significant changes were noted in the liver or pancreas.

The patient was advised to continue six-month follow-up with imaging studies to assess the resolution of biliary obstruction and to monitor for any potential recurrence of symptoms. He was also prescribed medications to manage his diabetes and fatty liver disease.

### 2.10 Patient perspective

The patient expressed gratitude for the prompt intervention and care provided during his hospital stay. He noted significant relief from the abdominal pain and jaundice after the surgical interventions, though he acknowledged the discomfort of recovery. He was concerned about the long-term management of his liver condition and was keen to adhere to medical advice for follow-up visits and lifestyle modifications.

### 2.11 Informed consent

The patient provided informed consent for all diagnostic and therapeutic interventions, including the endoscopic and surgical procedures, after a thorough explanation of the risks, benefits, and alternatives was provided.

## 3 Discussion

The case presented offers a complex scenario involving a 56-year-old male patient who was diagnosed with obstructive jaundice, potentially caused by a combination of bile duct stones, bile duct dilation, and a biliary-enteric fistula. The case was further complicated by the patient’s medical history, which included prior cholecystectomy and intestinal adhesion release, as well as a mild enlargement of the spleen. The patient’s symptoms were initially alleviated with supportive treatment, but the condition worsened, requiring more invasive interventions such as ERCP and laparoscopic surgery (1). This report raises important points regarding both the strengths and limitations of this case, the relevant medical literature, and the scientific rationale for the conclusions drawn.

One of the key strengths of this case report is the thorough diagnostic approach employed. A combination of imaging modalities, including ultrasound, CT scans, and MRCP, was used to confirm the diagnosis of obstructive jaundice and identify the possible causes, including bile duct stones and a biliary-enteric fistula (2). The integration of these diagnostic tools is in line with current best practices, as the use of advanced imaging can provide accurate visualization of biliary tract abnormalities, as supported by the literature (3). Furthermore, the patient’s surgical management—combining endoscopic procedures and laparoscopic surgery—demonstrates a comprehensive approach to addressing the multifactorial nature of the condition. This aligns with established treatment protocols for complex biliary diseases (4).

However, this case also has limitations. For one, the lack of pre-surgical biopsies or tissue samples to definitively confirm the etiology of the biliary-enteric fistula poses a challenge in establishing a clear causal relationship between the patient’s symptoms and the fistula itself (5, 6). Although imaging findings suggest the presence of the fistula, direct pathological confirmation would have strengthened the case. The absence of histopathological verification warrants further elaboration. Intraoperative biopsy was not pursued due to the significant technical challenges posed by dense inflammatory adhesions at the fistula site and concerns regarding iatrogenic injury to adjacent structures during dissection. Furthermore, the fistula’s macroscopic features—including its well-defined communication between the common bile duct and duodenum, active bile leakage, and surrounding fibrotic changes—were deemed pathognomonic under direct visualization. This aligns with standard surgical practice in similar emergent settings, where definitive anatomical identification and repair supersede histological sampling, particularly when imaging and operative findings are concordant (7, 8). Unlike Gibreel et al. (7) report on gallbladder-colostomy fistula patients requiring emergency open surgery, this case demonstrated that while ERCP decompression failed in the same patient, a minimally invasive obstruction relief was achieved through laparoscopic partial gastrectomy combined with Roux-en-Y gastrostomy, avoiding the significant trauma of open surgery. Moreover, compared to Agathis et al. (8) staged robotic approach (first addressing intestinal obstruction, then repairing fistulas), this case successfully completed adhesion lysis, fistula-related obstruction relief, and gastrointestinal reconstruction in a single laparoscopic procedure. This provides a novel clinical strategy for concurrent minimally invasive treatment of complex postoperative biliary fistulas with obstruction. In addition to the lack of pre-surgical biopsies, the collected intraoperative tissue samples of the fistula margin were small and had limited representativeness. Although HE staining confirmed chronic inflammation and fibrosis at the fistula site, the absence of more extensive tissue sampling (e.g., from the deeper layers of the fistula wall or the interface between the bile duct and duodenum) prevented definitive verification of the fistula’s etiology (e.g., whether it was directly related to the 2022 laparoscopic cholecystectomy or secondary to other inflammatory processes). Additionally, the patient’s medical history, including previous surgeries, could be a confounding factor in interpreting the progression of his current symptoms. The possibility that previous procedures might have contributed to the development of the biliary fistula or other complications is not fully explored in the report. As such, a more detailed exploration of the patient’s surgical history, particularly focusing on the outcomes and complications of prior surgeries, would have added depth to the analysis.

In comparing this case with the literature, several key similarities and differences arise. Previous studies have shown that obstructive jaundice often results from conditions such as choledocholithiasis, cholangitis, or biliary strictures. The patient’s symptoms, including jaundice, fever, and abdominal pain, are consistent with those described in the literature for biliary obstruction (9). However, the presence of a biliary-enteric fistula as a possible complication of prior surgery is a less common finding (10). Literature on this specific complication highlights its association with previous cholecystectomy and other biliary surgeries. This case, therefore, contributes to a broader understanding of the complications that can arise following abdominal surgeries and the importance of considering such factors in the diagnosis and treatment of biliary diseases.

However, while acknowledging the documented occurrence of biliary-enteric fistulas following biliary surgery (Table 1), our case offers distinct insights into diagnosis and management. The unique challenge stemmed from the combination of factors: a large fistula causing significant biliary leakage and obstruction, dense inflammatory adhesions obscuring anatomy consequent to multiple prior abdominal surgeries, and the concomitant presence of choledocholithiasis. Crucially, initial ERCP was not only diagnostic but also highlighted severe inflammatory stenosis at the fistula site, rendering endoscopic therapy insufficient and necessitating definitive laparoscopic surgical repair involving gastrectomy and Roux-en-Y reconstruction. This contrasts with many reported cases where fistulas were smaller, endoscopic management was successful, or the surgical history was less complex. Therefore, this case significantly contributes by detailing the diagnostic hurdles in complex post-surgical anatomy, demonstrating the critical role of ERCP in both diagnosis and triaging management, and outlining the specific laparoscopic surgical approach required when inflammation and adhesions preclude minimally invasive endoscopic resolution or less extensive surgery. The successful outcome underscores the importance of this tailored, step-wise approach in similar complex scenarios.

The causal relationship between the patient’s symptoms and the identified complications is complex. The primary causal factor for the patient’s obstructive jaundice appears to be the combination of bile duct stones and the biliary-enteric fistula (11). Previous studies suggest that biliary-enteric fistulas can develop as a result of chronic inflammation or complications from surgical procedures (12). In this case, the fistula likely resulted from the patient’s prior cholecystectomy and the associated changes in biliary anatomy. The inflammation at the site of the fistula further complicated the patient’s condition, as evidenced by the difficulty encountered during the ERCP. This provides strong evidence for a causal relationship between the surgical history and the current biliary obstruction, although more data on the patient’s surgical recovery and post-operative complications would help solidify this connection.

In terms of scientific rationale, the treatment choices were based on the understanding that biliary obstruction can lead to severe complications if not addressed promptly (13). Endoscopic interventions, including ERCP, are commonly used to manage biliary obstructions, and they are supported by the literature as effective first-line treatments for such conditions (14). The laparoscopic approach, involving a gastrectomy and Roux-en-Y gastrojejunostomy, was an appropriate next step given the patient’s worsening condition and the potential for a long-term solution to the biliary obstruction. The use of laparoscopic techniques, while more invasive, is supported in cases of complex biliary pathology where non-surgical interventions may not suffice (15).

Initially, ERCP was attempted for both diagnostic and therapeutic purposes. However, severe inflammatory stenosis and a significant duodenal-biliary fistula were encountered, precluding successful endoscopic intervention. In light of the patient’s clinical presentation—including abdominal pain, biliary infection, and pneumobilia—a multidisciplinary consensus was reached to proceed with laparoscopic Roux-en-Y gastrojejunostomy. This approach was selected to effectively alleviate symptoms and improve long-term outcomes, particularly given the concern for ongoing biliary infection which rendered biliary-enteric anastomosis less favorable. The postoperative course confirmed the efficacy of this strategy, with resolution of symptoms and stable clinical follow-up.

The case also presents a unique set of circumstances, with the potential for a biliary-enteric fistula complicating the presentation of obstructive jaundice (16). The patient’s previous surgeries, including cholecystectomy, likely predisposed him to the formation of this fistula. While such complications are rare, they are documented in the literature, and this case serves as a reminder of the complexities involved in managing patients with a history of abdominal surgeries (17).

From a clinical perspective, this case highlights several important lessons. First, it underscores the need for careful post-surgical follow-up, especially in patients who have undergone complex biliary or abdominal surgeries. It is crucial to monitor for late complications such as biliary-enteric fistulas, which may present with non-specific symptoms like jaundice and abdominal pain. Additionally, it reinforces the importance of a multidisciplinary approach in managing complex cases, particularly those involving multiple interventions like endoscopy and surgery. Given the potential for recurrence of symptoms or complications, ongoing monitoring is essential, and patients should be educated about the signs of potential issues following biliary surgery.

## 4 Conclusion

The case of a 56-year-old male with obstructive jaundice, complicated by bile duct stones and a biliary-enteric fistula, underscores the importance of a comprehensive diagnostic approach and tailored therapeutic interventions. The patient’s condition was successfully managed through a combination of ERCP and laparoscopic surgery. The use of advanced imaging techniques (ultrasound, CT, MRCP) and limited intraoperative tissue sampling proved essential in confirming the diagnosis of biliary-enteric fistula. Although the small size of the fistula tissue samples prevented definitive verification of the etiology, the pathological findings of chronic inflammation and fibrosis supported the inflammatory nature of the fistula, guiding the selection of surgical treatment strategies. This case emphasizes the need for careful follow-up in patients with complex biliary conditions, particularly those with a history of abdominal surgeries. It highlights the importance of a multidisciplinary approach to ensure effective management of multifactorial diseases. For future research, further studies into the long-term outcomes of patients with biliary-enteric fistulas and the role of minimally invasive techniques in managing such cases are recommended. This case provides valuable insights for clinical practice, particularly in the management of post-surgical biliary complications, and reinforces the need for vigilance in the follow-up care of patients with complex biliary diseases.

## Data Availability

The original contributions presented in the study are included in the article/Supplementary material, further inquiries can be directed to the corresponding authors.
